# Nucleophosmin in Its Interaction with Ligands

**DOI:** 10.3390/ijms21144885

**Published:** 2020-07-10

**Authors:** Ilaria Cela, Adele Di Matteo, Luca Federici

**Affiliations:** 1 Center for Advanced Studies and Technology (CAST), University of Chieti “G. d’Annunzio”, Via Polacchi, 66100 Chieti, Italy; ilaria.cela@unich.it; 2 Department of Medical, Oral and Biotechnological Sciences, University of Chieti “G. d’Annunzio”, Via dei Vestini 31, 66100 Chieti, Italy; 3 Institute of Molecular Biology and Pathology (IBPM) of the CNR, c/o “Sapienza University of Rome, Piazzale Aldo Moro 5, 00185 Roma, Italy; adele.dimatteo@cnr.it

**Keywords:** nucleophosmin, B23, acute myeloid leukemia, AML, protein–protein interactions, protein–nucleic acids interactions, cancer

## Abstract

Nucleophosmin (NPM1) is a mainly nucleolar protein that shuttles between nucleoli, nucleoplasm and cytoplasm to fulfill its many functions. It is a chaperone of both nucleic acids and proteins and plays a role in cell cycle control, centrosome duplication, ribosome maturation and export, as well as the cellular response to a variety of stress stimuli. NPM1 is a hub protein in nucleoli where it contributes to nucleolar organization through heterotypic and homotypic interactions. Furthermore, several alterations, including overexpression, chromosomal translocations and mutations are present in solid and hematological cancers. Recently, novel germline mutations that cause dyskeratosis congenita have also been described. This review focuses on NPM1 interactions and inhibition. Indeed, the list of NPM1 binding partners is ever-growing and, in recent years, many studies contributed to clarifying the structural basis for NPM1 recognition of both nucleic acids and several proteins. Intriguingly, a number of natural and synthetic ligands that interfere with NPM1 interactions have also been reported. The possible role of NPM1 inhibitors in the treatment of multiple cancers and other pathologies is emerging as a new therapeutic strategy.

## 1. Introduction

**Figure 1:**
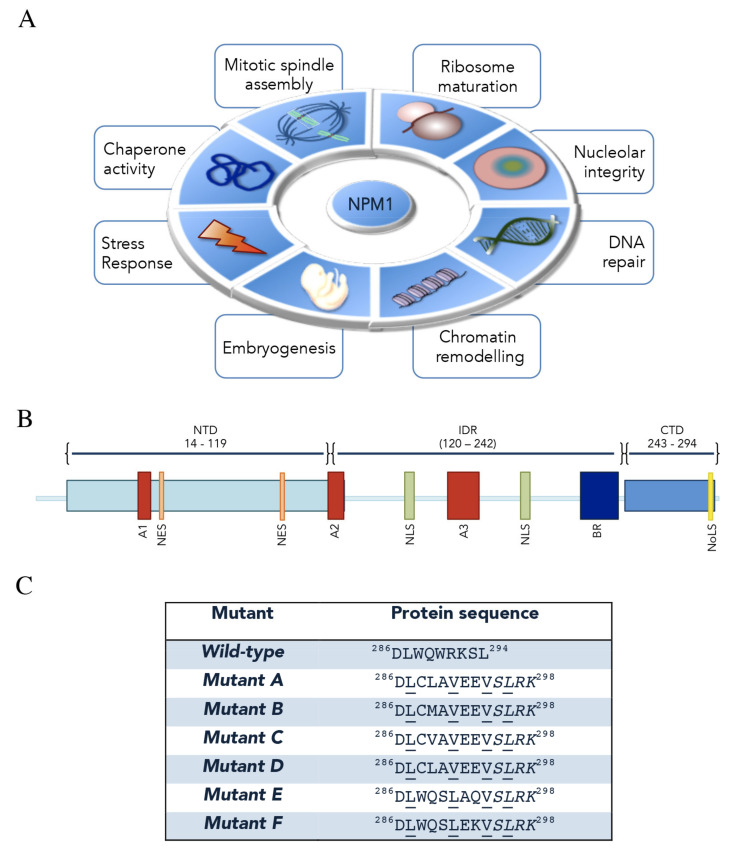
Nucleophosmin (NPM1) functions, structural organization and acute myeloid leukemia (AML) mutations. (**A**) The main functions played by NPM1 through its interactions with proteins and nucleic acids are represented. (**B**) NPM1 is composed of a N-terminal domain (NTD) preceded by a methionine-rich short sequence. NTD contains two nuclear export signals (NES) and a short acidic tract (A1). NTD is followed by an intrinsically disordered region (IDR) which contains two acidic tracts (A2 and A3), a bipartite nuclear localization signal (NLS) and a basic region (BR). Finally, a positively charged C-terminal domain (CTD), which contains the nucleolar localization signal (NoLS), is present. (**C**) Heterozygous AML-associated mutations cause the expression of a protein that is longer by four residues and has a different sequence in the last seven. A novel NES appears in all mutants (underlined residues). Here, the C-terminal sequences of wild-type and the most common mutants are shown.

Nucleophosmin (NPM1, also known as B23) is an abundant and multifunctional phosphoprotein mainly found in nucleoli [1]. Despite its predominant nucleolar localization, NPM1 is able to rapidly shuttle between nucleus and cytoplasm to exert its many and different functions in several cellular processes, which have been extensively reviewed recently [2,3,4,5,6]. Among them, NPM1 is implicated in: (i) ribosome assembly and export [7,8]; (ii) centrosome duplication and cell cycle control [9,10]; (iii) DNA replication and repair [11,12]; (iv) histone- and protein-chaperone activity [13,14]; (v) response to stress stimuli [15]; (vi) embryogenesis [1]. Intriguingly, a key role of NPM1 in the organization of the granular component of nucleoli through the formation of liquid–liquid phase separation (LLPS) was recently uncovered [16,17] (Figure 1A).

The human *npm1* gene resides on chromosome 5q35 locus and consists of 12 exons encoding for at least three isoforms derived from alternative mRNA splicing [18,19]. NPM1.1, or simply indicated as NPM1, is the most represented variant and corresponds to the longest transcript, coding for a 294-aa protein (37 kDa) expressed ubiquitously in all tissues [1]. The second variant (NPM1.2) corresponds to a 265-aa protein which lacks exon 8 [20,21]; the third protein isoform (NPM1.3) was shown to be expressed at low levels within nucleoplasm and not nucleoli, due to lack of the last 35 amino-acids in the C-terminus [22,23]. Little information is available for NPM1.2 and NPM1.3 while the best-characterized isoform, NPM1, is the topic of this review.

Structurally, NPM1 shows a modular architecture comprising distinct, although partially overlapping, functional regions that mediate multifaceted roles in different cellular events [2,24] (Figure 1B). The N-terminal region is highly conserved among all members of the nucleophosmin/nucleoplasmin family of histone chaperones, to which NPM1 belongs [25]. This region comprises a short N-terminal methionine-rich unstructured sequence of unknown function and the N-terminal “core” domain (NTD—Res: 14–119). This is formed by eight antiparallel beta-strands forming a jellyroll barrel and is mainly, but not exclusively, responsible for NPM1 binding to protein partners, while it is also crucial for self-oligomerization [24,26]. Indeed, within cells, a substantial fraction of NPM1 behaves as an oligomer where five NPM1 monomers tightly associate to form a crown-shaped pentamer [21,27]. Furthermore, two pentamers of NPM1 may interact in a head-to-head fashion forming a decamer, arranged so that each monomer of the pentameric ring contacts only a single monomer of the other pentamer [27,28,29]. Notably, the monomer–pentamer equilibrium is fine-tuned through a multisite phosphorylation mechanism, which induces the unfolding of NPM1 monomer, and via intermolecular interactions that conversely promote NPM1 oligomer assembly [26,30]; importantly, this equilibrium might be strictly associated with modulation of NPM1 functions and subcellular localization [6,30]. Within the NPM1 N-terminal domain, two leucine-rich nuclear export signals (NES) are included (residues 42–49 and 94–102, respectively) and responsible for NPM1 cytoplasmic shuttling mediated by exportin-1/CRM1 [10,31]. The N-terminal core domain also contains a first acidic tract, termed A1 (residues 36–39) [27]. NTD is followed by a central intrinsically disordered region (IDR) in which two highly acidic stretches composed of several consecutive aspartic or glutamic acidic residues, namely A2 (residues 120–132) and A3 (residues 161–188) tracts, are present [26,32,33]. These two segments, together with the acidic A1 tract within the “core” domain, are thought to be functional for histone binding, possibly by mimicking the negative charge of DNA/RNA, in order to facilitate nucleosome assembly and chromatin remodeling (Figure 1B) [34,35]. In addition, before and after the A3 tract, a bipartite nuclear localization signal (NLS) is present (residues 152–157 and 191–197, respectively) [24]. In between the A3 acidic region and the C-terminal domain (CTD), NPM1 contains a markedly basic segment of over 50 residues that have been suggested to facilitate NPM1 interactions with nucleic acids, working in concert with the C-terminal domain (CTD, Figure 1B) [17,36,37]. The latter consists of a right-handed three-helix bundle that is stabilized by a hydrophobic core mainly formed by five conserved aromatic residues (F268, Y271, F276, W288, W290). CTD contains several exposed lysine and arginine residues, which account for its positively charged surface [38,39]. The region encompassing the two tryptophan residues W288 and W290, within the CTD, has been ascribed to form an atypical nucleolar localization signal (NoLS), specific to NPM1 [39,40]. Importantly, this domain is unique to NPM1 as compared to other members in the nucleophosmin/nucleoplasmin family [39] and its deletion, or alterations at the two key tryptophan residues that cause domain unfolding, compromise both nucleic acid binding and nucleolar localization [38,41,42] (Figure 1B). As will be described in the following sections, such mutations are typically found in acute myeloid leukemia (AML) patients (Figure 1C).

## 2. Role in Cancer

Much of the interest in NPM1 arises from its crucial implication in tumorigenesis. This is related to frequent alterations of its expression levels or chromosome translocations or mutations involving its gene [1]. Notably, NPM1 is overexpressed in a wide spectrum of solid human cancers including prostate [43], ovaries [44], nonsmall cell lung [45], liver [46], thyroid [47], colon [48], pancreas [49], as well as glioma [50], glioblastoma [51] and astrocytoma [52]. It has been highlighted that, in some cases, NPM1 increased expression correlates with the mitotic index and with the stage of tumor progression [1,53]. Even if the role of NPM1 overexpression in cancerogenesis is far to be fully understood, various scenarios have been proposed including the association of its overexpression with the upregulation of ribosome biogenesis and protein synthesis [54], but also with the stimulation of DNA repair following oncogene activation and with reduced apoptotic response [15,55].

*Npm1* alterations are cardinal to various hematological malignancies [3,4,6]. Here, chromosomal translocations, deletion and several types of mutations have been found and widely investigated. In hematological tumors, the *npm1* gene is frequently combined with other genes giving rise to fusion products that often retain only the NPM1 N-terminal oligomerization domain. This results in a reduction of NPM1 wild-type content and in the nucleoplasmic or partially cytosolic dislocation of the resulting fused proteins, due to the absence of the NPM1 NoLS region in the chimeras [4]. In anaplastic large cell lymphoma (ALCL), the NPM1-ALK (Anaplastic Lymphoma Kinase) translocation t(2;5)(p23;q35) causes the expression of a chimera in which the NTD of NPM1 is fused with the ALK kinase domain. The resulting chimera promotes ALK dimerization and its constitutive activation, which contributes to ALCL tumorigenesis [56]. In a subset of acute promyelocytic leukemia (APL) patients, as a consequence of the t(5;17)(q35;q31) translocation, the NPM1 N-terminal portion is fused with the DNA-binding domain of RARα (retinoic acid receptor α), altering the transcriptional activity of the latter [57]. Another chromosomal translocation, t(3;5)(q25;q35), produces the chimeric protein NPM1-MLF1 (myeloid leukemic factor 1) which is associated with the onset and the multistep progression of myelodysplastic syndrome (MDS) into AML [58]. A more recent translocation, t(5;19)(q35;p13), was observed in lymphoproliferative disorders (LPDs) and consists in the fusion of almost full length NPM1 with the catalytic domain of TYK2 (protein tyrosine kinase 2), whose kinase activity results constitutively activated, in a way similar to what reported for ALK-NPM1. TYK2 activation is then responsible for the activation of several downstream effectors, such as STAT proteins, thus promoting cell survival [59,60]. Another novel genomic rearrangement, resulting in the chimeric protein NPM1-HAUS1 (Augmin-Like Complex Subunit 1), has been identified in AML patients [61], while an interstitial deletion of 5q associated with NPM1 haploinsufficiency has been observed in myelodysplastic syndromes (MDS) [62] but also found in AML [63] and in T-cell acute lymphoblastic leukemia (T-ALL) [64].

The *Npm1* gene, as mentioned before, is also the target of several mutations that always occur in a heterozygous way [65]. AML mutations map mostly within exon 12 of the gene and typically consist of 4-base pair frameshift duplication or insertion [39,66]. More than 50 different mutations have been described however, in all cases, the consequences in the protein sequence are very similar, resulting in a protein containing four additional amino-acids and a different sequence in the last seven residues [65] (Figure 1C). In all cases, one or both of the two key tryptophan residues forming the NoLS are replaced, inducing (i) the total unfolding or massive destabilization of the whole C-terminal region of NPM1 and (ii) the impairment of NPM1 interaction with nucleic acids in the nucleoli [38,42]. Importantly, the acquired C-terminal sequence corresponds to a new NES signal which is added to the other two naturally present within the NPM1 sequence (Figure 1C). This, in addition to the NoLS disruption, causes the aberrant cytoplasmic delocalization of mutated NPM1, which for this reason is also commonly referred to as NPM1c+, where c+ stands for “cytoplasmic positive” [65]. Since NPM1c+ retains its N-terminal domain unaltered, it maintains the ability to oligomerize with the wild-type form of NPM1, leading to the delocalization of the majority of the latter. As a consequence, only a small fraction of NPM1 remains within the nucleoli of leukemic blasts, which is anyway sufficient to fulfill the crucial protein’s nucleolar functions [41,65]. Indeed, it was shown that double *npm1* knockout-out mice die at the early embryonic stage due to massive hematopoietic defects [67].

NPM1 interacts with a number of protein partners, mostly with its N-terminal domain (see below), and the contribution that NPM1c+ plays in the alteration of different cellular processes, potentially contributing to leukemogenesis, has been widely investigated. The main mechanism appears to consist of the cytosolic dislocation of several NPM1 interactors, through their interaction with the unaltered NPM1c+ NTD. Among the best-characterized dislocated partners are the tumor suppressor p14ARF (Alternate Reading Frame protein product of the CDKN2A locus) [68], APE1 (Apurinic/apyrimidinic endonuclease 1) [69], the c-Myc ligase Fbw7γ (F-box/WD repeat-containing protein 7) [70] and the transcription factor PU.1 [71]. A cytosolic NPM1c+-mediated inhibition of caspase activities and of HAUSP (Herpesvirus-associated Ubiquitin-specific Protease), a PTEN deubiquitinating enzyme, has also been reported [72,73]. Overall, these interactions may result in inhibition of differentiation, apoptosis and DNA repair [4,6].

Mutations in the exon-12 of the *npm1* gene are the most frequent genetic lesions observed in AML patients with normal karyotype (AML-NK), accounting for almost 30% of all AML patients [4,74]. In 2016, AML with mutated *npm1* has been defined as a distinct entity in the World Health Organization (WHO) classification of hematopoietic malignancies [75]. Until today about 50 mutations in the exon-12 of *npm1* have been reported, all between nucleotide positions 861 and 894 [3,5,39,76]. The most common *NPM1* mutation is type-A, accounting for about 75–80% of the patients, and consisting of a “TCTG” tetranucleotide tandem duplication [65,77]. Type-B (“CATG” insertion) and type-D (“CCTG” insertion) mutations are less common, harbored by 10% and 5% of NK-AML patients, respectively [5,65,78]. Other exon-12 lesions are rare and account for less than 1% of NK-AML patients [3] (Figure 1C). Notably, it has been recently suggested that these lesions could arise from replication slippage errors primed by altered terminal deoxynucleotidyl transferase (TdT) activity through N-nucleotide addition [79]. This mechanism was also proposed for *FLT3*-ITD (internal tandem duplication) mutations [80], which are twice as frequent in *NPM1*-mutated AML as compared to AML with wild-type *NPM1* [3,65]. These observations reinforce the mechanistic cooccurrence of these two mutations and the idea that *NPM1* mutations likely precede those of *FLT3*-ITD [3,81]. Conversely, *NPM1* mutations are thought to be secondary to those occurring on the *DNMT3A* gene, coding for a DNA methyltransferase, that appear and persist already in the preleukemic hematopoietic stem cells (HSCs) [78,82], thus suggesting that *NPM1* mutations are later drivers in leukemogenesis [83]. In addition to these notable NPM1-concomitant mutations, lesions in *npm1* gene have been reported to cooccur also with those in *idh1* (Isocitrate dehydrogenase), *idh2* and *tet2* (Methylcytosine dioxygenase), all encoding for epigenetic modifiers [78,84]. However, *npm1* mutations have also been observed to be mutually exclusive with others, such as tandem duplication of *MLL* (Mixed lineage leukemia) gene, but also with mutations in *runx1* (Runt-related transcription factor 1), *cebpa* (CCAAT/enhancer-binding protein alpha) and *tp53* genes [85]. Finally, also *npm1* non-exon-12 mutations have been observed. In a recent work by Nachmani and colleagues [86], germline mutations of the *npm1* gene have been associated with the etiogenesis of the ribosomopathy dyskeratosis congenita: these lesions consist of a missense mutation or an in-frame-deletion within the A3 acidic region of NPM1 (see next paragraph) [86]. Interestingly, the inspection of the Cancer Genome Atlas revealed the presence of mutations in the A3 tract also in a subset of solid cancers [86]. The role that these mutations may play in different tumors deserves further investigations.

## 3. Interactions

### 3.1. Nucleic Acids

**Figure 2:**
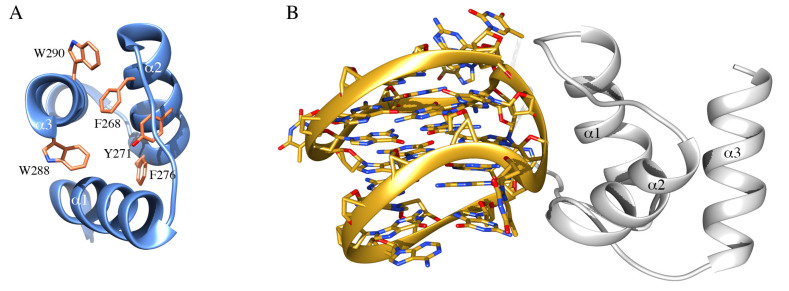
NPM1 C-terminal domain and interaction with nucleic acids. (**A**) The structure of the NPM1 C-terminal domain, consisting of three helices forming a bundle, is represented in ribbon. In sticks are highlighted residues forming the hydrophobic core of the three-helix bundle, including W288 and W290, that are affected by AML-related mutations. (**B**) Structure of the complex between NPM1 CTD (white ribbon) and the G-quadruplex region from the c-MYC promoter (gold ribbon). The phosphate backbone of the G-quadruplex is engaged by a positively charged groove between helices 1 and 2.

Due to its predominant localization in the granular region of nucleoli, where the maturation of ribosomal particles occurs, a possible role for NPM1 in nucleic acid binding was soon hypothesized and corroborated by in vitro experiments. First, it was shown that NPM1 was able to interact with both DNA and RNA [87,88] and the interaction was mapped to the last 70 residues of the reference sequence, comprising the CTD and a portion of the flanking basic segment belonging to the IDR (Figure 1B) [24]. At this stage, a preference for single-stranded over double-stranded DNA samples was detected but no sequence requirements for binding were assessed [24,88]. Interestingly, a ribonuclease activity exerted at the level of 28S rRNA maturation was detected, suggesting for NPM1 a role in ribosome maturation besides its role in ribosome export from nucleoli [89]. The discovery of AML-related mutations in the CTD of NPM1 prompted a renewed interest in this domain and its activities by many groups, including ours. Initial structural studies showed the domain to consist of three helices arranged in a bundle [38] (Figure 2A) that may retain residual structure also in the denatured state [90,91]. As to the function of the domain, an important observation was the first identification of a specific DNA sequence recognized by NPM1, at the promoter of the SOD2 gene [92]. This sequence was suggested to form a hairpin characterized by the presence of ten consecutive guanine nucleotides in the hairpin loop (G10-loop). Subsequently, while testing structural features of the G10-loop for high-affinity recognition by NPM1 CTD, we could demonstrate that the actual structure adopted by this sequence in vitro, and possibly in vivo, is that of a parallel G-quadruplex and not of a hairpin loop [36].

Indeed, NPM1 CTD demonstrated a higher binding affinity for G-quadruplex DNA as compared with single or doubled stranded DNA, even though being able to recognize any oligonucleotide tested. Furthermore, an important contribution to the overall affinity by the basic region belonging to the central IDR of NPM1 and immediately preceding the CTD, was envisaged [36] and later confirmed by different groups [93,94]. The structure of a larger NPM1 truncated version, encompassing both the CTD and the flanking basic region, in complex with a prototypical parallel G-quadruplex DNA from the c-MYC promoter was investigated by NMR [95] (Figure 2B). The CTD three-helix bundle engages the phosphate backbone of the G-quadruplex with a groove between helices 1 and 2. Several lysine residues face this groove and mutational studies indicated that the contribution of all of them is necessary for high-affinity recognition [37]. Most interestingly, in the complex structure, the flanking basic tail was shown not to form stable contacts with the G-quadruplex [95]. However, site-directed mutagenesis of lysine residues in the tail, coupled with kinetic analysis of the interaction and MD simulations, suggested a model whereby the tail provides long-term electrostatic interactions to facilitate the encounter between NPM1 CTD and the G-quadruplex but also takes part to the complex formation, albeit only transiently and in a time-scale not detectable by NMR [37]. Interestingly, it was also observed that acetylation of lysine residues located both at the interface with the G-quadruplex (K250 and K257) or in the basic tail (K229 and K230) is equally able to displace the protein from nucleoli [96]. The appreciation of NPM1 CTD affinity for G-quadruplex structures in vitro raised the question as to whether specific G-quadruplexes are bound by the protein in vivo. Interestingly, a number of putative G-quadruplex sequences were found at the nontemplate strand of the rDNA gene [97]. Investigation of these sequences confirmed that they are indeed recognized by NPM1 both in vitro and in vivo [42]. Furthermore, it was shown that (i) G-quadruplex ligands are able to displace wt-NPM1 from nucleoli, (ii) the CTD of NPM1c+ is unfolded and loses its ability to bind G-quadruplexes in vitro and (iii) reinsertion of the two tryptophan residues in the context of the mutated sequence restores both G-quadruplex binding [42] and nucleolar localization [41]. Taken together these studies provided a correlation between the correct folding of the CTD, which is totally or partially lost in AML-related mutants, and the nucleolar retention of the protein.

The studies cited above were focused on DNA binding but NPM1 is also known to bind RNA. In 2014 a comprehensive analysis of NPM1 rRNA binding properties was provided [98]. First, it was shown that NPM1 interacts preferably with 28S, 5.8S and 5S rRNA as compared to 18S rRNA. Then, the structural requirements for the interaction were investigated assessing that (i) the basic portion of the IDR is able to aspecifically recognize rRNA with low affinity and coadjuvanate CTD in rRNA binding and (ii) phosphorylation at specific sites in the basic portion of IDR abolishes NPM1 rRNA binding activity. Furthermore, the IDR also contains two markedly acidic tracts and intra- or intermolecular interactions with the basic portion of the same domain and with the CTD were suggested, adding complexity to the NPM1–rRNA interaction [98]. Overall, the role in the nucleic acid binding of the IDR basic region that emerges from this study is not dissimilar from what has been envisaged in the case of several transcription factors and linker histone H1: while the CTD preferably binds structured DNA/RNA, the flanking tail may both help recognition of specific structures/sequences and stabilize the final complex through direct albeit aspecific binding [99].

It is worth noting that the NPM1 RNA-binding ability is not limited to rRNA. Indeed, a recent seminal paper reported a comprehensive analysis of the RNA bound by NPM1 in vivo obtained through HiTS-CLIP analysis [86]. This analysis identified rRNA, noncoding, intergenic, tRNA and protein coding sequences. However, the most represented class of RNA molecules found interacting with NPM1 was that of snoRNAs, in particular those belonging to the C/D box class. These snoRNAs are known to work in concert with fibrillarin to mediate specific ribosomal RNA 2′-O-methylation (2′-O-Me). Indeed, loss of NPM1 resulted in a significant reduction of 2′-O-Me levels at five specific rRNA sites. NPM1 was shown to independently bind both fibrillarin, the methylating enzyme, and snoRNAs which provide rRNA substrate specificity. Under this light, NPM1 would be essential to trigger the formation of a functional ternary complex and its loss or cytoplasmic delocalization would impair proper snoRNP formation and 2′-O-Me in cells. Interestingly enough, the same study identified two novel germline mutations in the NPM1 gene in patients suffering from dyskeratosis congenita, and thus linked impaired 2′-O-Me at multiple levels to this ribosomopathy, characterized by bone marrow failure and associated symptoms. The two novel mutations are both located at the acidic tract A3 (D178H and D180del, Figure 1B) and were shown to affect snoRNA binding and loading into C/D box snoRNPs [86]. It is also worth mentioning that inspection of the Cancer Genome Atlas highlights the presence of mutations in the acidic repeats in a number of cancers of different histological origin, suggesting an important role for NPM1 in epitranscriptome and that aberrant or altered 2′-O-Me may be pathological. How mutations at the acidic tracts of the IDR may affect snoRNAs (and possibly other RNAs) recognition by NPM1 is a question that must be absolutely answered through structural studies.

### 3.2. Proteins and Peptides

NPM1 is very promiscuous in its interaction with protein partners. Indeed, proteins already shown to interact with NPM1 are several dozens and the list is growing every year with new evidence (https://thebiogrid.org/110929/summary/homo-sapiens/npm1.html). Collectively, these interactions implicate a direct or indirect involvement of NPM1 in processes including (i) DNA replication, transcription and repair, (ii) cell cycle control, (iii) ribosome biogenesis, (iii) nuclear–cytoplasmic shuttling of viral proteins and viral replication, (iv) apoptosis, (v) stability and splicing of mRNA, (vi) protein modification and degradation, (vi) mitotic spindle, centromeres and cytoskeleton binding. All these aspects of NPM1 functions have been recently covered by a number of excellent reviews [2,3,4,5,6,54]. Here, we focus on what is known as to how NPM1 recognizes its protein partners from a structural standpoint.

One first evidence of NPM1 protein binding properties emerged soon after its discovery and is related to its activity as a histone chaperone. Indeed, it was shown that NPM1 is able to bind denatured protein substrates in vitro and to impede or to retard their aggregation, which is a hallmark of chaperone activity [13]. Furthermore, it was shown that NPM1 acts as a histone chaperone in the nucleolus since it was observed how this protein is able to assemble nucleosomes and to decondense sperm DNA [14]. In addition, direct binding of histones H2A, H2B, H3 and H4 was shown; interestingly, while H3 and H4 are weakly bound by the NPM1 core region alone, which comprises the A1 acidic tract, the presence of the A2 and A3 acidic tracts was demonstrated to be a prerequisite for H2A and H2B binding [14]. Later on, NPM1 was also found able to bind linker histone H1 and to efficiently deposit it on dinucleosomal templates; once again this activity was associated with the function of the acidic tract A2 (120–132 residues) [34]. These data were integrated by the discovery of the association of NPM1 with several ribosomal proteins, including RPL5 [31], RPS9 [100] and RPL23 [101]. These studies, together with others showing that NPM1 is involved in the processing of pre-rRNA to mature 28S [102] and that blocking NPM1 nuclear–cytoplasmic trafficking inhibits the export of ribosomal subunits [7,54], established this protein as a possible dual chaperone for both nucleosomes and ribosomes.

Even earlier than studies aimed at clarifying physiological protein–protein interactions mediated by NPM1, its involvement in a number of interactions with viral proteins was discovered. Studies include, but are not limited to, interactions with Tat and Rev proteins from HIV [103], hepatitis B core protein [104] and adenovirus basic core proteins [105]. These and other studies have clarified that NPM1 is involved in different stages of the viral life cycle ranging from the nuclear import of viral proteins to final assembly [106]. Importantly, studies from NPM1 interactions with viral proteins started to shed light on these associations from a structural point of view: the N-terminal core domain either in cooperation with the central acidic tracts or not is the main region devoted to NPM1 protein associations. Indeed, several studies identified the NPM1 NTD as structurally responsible for the binding of viral proteins, assisting them in their transport to and localization in the nucleus and nucleoli [103,107,108,109]. As such NPM1 has been proposed as a target for the treatment of several viral infections [106].

**Figure 3:**
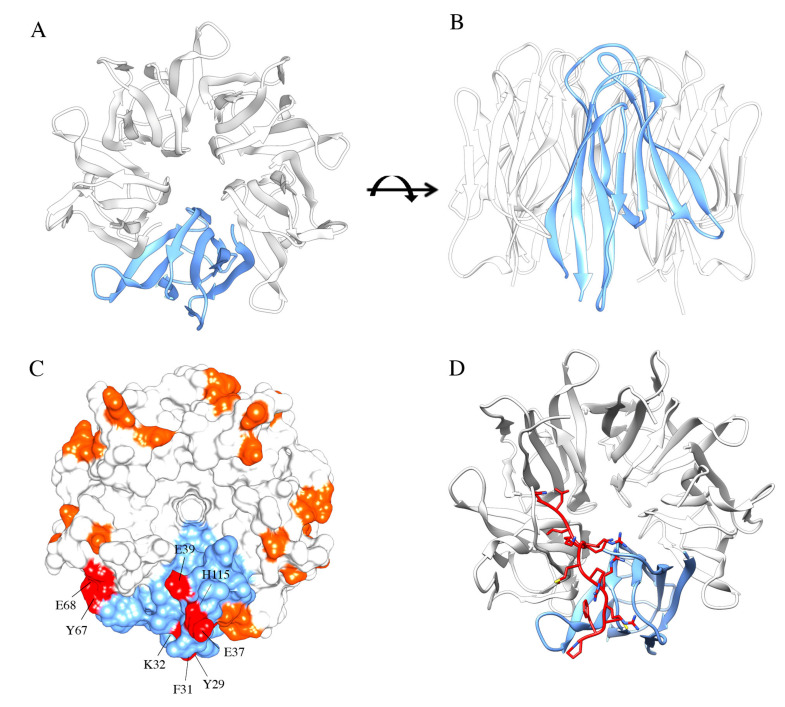
NPM1 N-terminal domain and interaction with arginine-rich motifs. NPM1 N-terminal domain (NTD) consists of eight antiparallel beta-strands forming a jellyroll barrel. Monomers associate to form a homopentamer, here represented from above (**A**) and in front view (**B**). (**C**) Arginine-rich motifs, often predicted to be the NoLS in protein partners, interact with residues of two adjacent monomers. Residues identified from the NMR analysis of the NPM1-p14ARF complex are highlighted in this surface representation. (**D**) Docking analysis of the interaction between NPM1 NTD and the NoLS peptide from Fbw7γ. The peptide adopts an extended conformation and engages two adjacent monomers.

Among NPM1 protein interactors, one of the most characterized is the human tumor suppressor p14ARF, and its murine homolog p19ARF [102,110]. In unstressed cells, NPM1 and p14ARF colocalize in the nucleolus where they are found in high molecular weight complexes. In response to oncogenic signals, both proteins are dispersed throughout the nucleoplasm wherein p14ARF exerts its tumor-suppressive function by promoting p53 stabilization through interaction with HDM2 [110]. The first investigations identified the region encompassing the first 192 N-terminal residues of NPM1, including the whole oligomerization “core” domain and the acidic tracts, as crucial for binding the p14ARF N-terminus region [102,111] (Figure 3A–C).

Further structural studies started to uncover the specific residues involved in this association, initially by considering a short linear ARF-derived peptide, corresponding to the very first N-terminal amino acidic residues of both p14ARF and p19ARF proteins, composed of at least two arginine residue stretches interspersed with hydrophobic residues [26,112]. Notably, mutagenesis assays revealed that these arginine residues were fundamental contributors to the interaction with the murine NPM1 N-terminal segment (1–130 residues); then, other short peptides, all sharing these R-rich motifs, and deriving from other NPM1 interactors, were tested for their interaction with NPM1 NTD. All these interactors were recognized with similar affinities and the binding surface was mapped in an acidic region formed at the interface between NPM1 protomers and on the top of the pentamer [26]. In the same work, it was pointed out that these R-rich motifs promoted the stabilization of the NPM1 pentamer, counteracting the otherwise repulsive interactions between A1 and A2 acidic tracts of adjacent protomers. A complex equilibrium between folded pentamers and partly or totally unfolded monomers, mainly influenced by the phosphorylation status of several residues in the core domain, was envisaged [26,30]. More recently, these studies were integrated by analyzing the interaction in the context of full-length human p14ARF [113]. It was shown, by NMR, that p14ARF is completely unfolded and tends to associate, through its N-terminal end, in grossly insoluble aggregates. Conversely, it associates with NPM1 in large soluble molecular weight assemblies. Importantly, a novel region on p14ARF that interacts with NPM1 N-terminal domain was identified in the C-terminal tract of p14ARF. This region is also rich in arginine residues and predicted to constitute the p14ARF NoLS [113,114]. These findings were consistent with the ability of NPM1 to recognize putative NoLS sequences in its interactors (see below) [33,115]. An extended region on the external surface of each NPM1-Nter monomer involved in the interaction with the p14ARF NoLS was identified, composed of several nonadjacent residues (Y29, F31, K32, E37, E39, Y67, E68, H115) [26,113] (Figure 3C). Altogether these data highlighted a complex scenario for the NPM1-p14ARF interaction where p14ARF has a tendency to assemble in insoluble homo-oligomers, while NPM1 and p14ARF form soluble supramolecular complexes that involve NPM1 core domain and at least two distinct regions at both ends of p14ARF, suggesting a mechanism through which NPM1 may sequester p14ARF in the nucleoli [113,116]. Since p14ARF is one of NPM1 interactors that are displaced to the cytoplasm in *NPM1*-mutated AML [68] and given that p14ARF is often altered in several cancers [117], the NPM1 surface that interacts with p14ARF has been proposed as a target for interfering small molecules to be used in the treatment of AML or possibly other cancers [118].

These and other evidence suggested the general idea that NPM1 recognizes many of its protein partners through short linear motifs, enriched in positive residues that constitute a NoLS. This idea was recently investigated using the NPM1 interaction with the tumor suppressor Fbw7γ as a model system [115]. Fbw7γ is an E3-ubiquitin ligase whose nucleolar localization and stabilization are strictly dependent on nucleolar NPM1 [70,115]. Notably, Fbw7γ has two other isoforms, Fbw7α and Fbw7β, that do not display nucleolar localization. Starting from these premises, putative NoLS were searched on the Fbw7γ sequence through the NoD algorithm [114,119] and a short peptide containing two clusters of three positively charged residues each, separated by a hydrophobic residue, was identified in the N-terminal end of Fbw7γ [115]. Importantly, the N-terminal ends of Fbw7α and Fbw7β are different and not predicted to be a NoLS. The interaction between NPM1 “core” domain and the Fbw7γ predicted NoLS was then verified in vitro through fluorescence spectroscopy. The same algorithm was then used to identify a putative NoLS in other NPM1 interacting partners, i.e., CENP-W and Tat, and again the interaction was successfully tested in vitro, providing general relevance to the concept that NoLS are specifically bound by NPM1, possibly explaining the nucleolar localization of the proteins that carry them [115]. A number of negatively charged residues in the NPM1 core domain (D36, E37, D39, E93 and E121), identifying a surface that overlaps with the one that interacts with p14ARF, were further investigated. Extended alanine-scanning mutagenesis on these residues, alone and in combinations, coupled to docking analysis and molecular dynamics simulations, allowed to observe that (i) the peptide binds this NPM1 surface in an extended conformation (Figure 3D), (ii) no single NPM1 residue may be considered a hotspot for the interaction, (iii) indeed, at least three negative charges have to be removed to start affecting the binding energy and (iv) the same NPM1 surface is responsible for the recognition of all tested NoLS peptides (Figure 3D). Furthermore, molecular dynamics simulations for the interactions suggested that the peptide populates, along the trajectory, different conformations that engage different negatively charged residues of NPM1 in the various poses [115]. It is noteworthy to observe that NoLS peptides are different in length and in the spacing of positively charged residues, but they are all recognized with similar affinity. Data suggest that all peptides find within the NPM1 core domain a large negatively charged surface provided with many anchor points and will shift rather freely within this surface, according to their particular distribution of positive charges. We hypothesize that such mechanism may be at the base of NPM1 ability in recognizing and binding target sequences from a plethora of different protein partners, a feature that is at the heart of NPM1 behavior as a “nucleolar hub” [115].

### 3.3. Role of NPM1 in Nucleolar Liquid–Liquid Phase Separation

The nucleolus is the site of ribosome biogenesis and an important cellular stress sensor. It is a membrane-less organelle (MLO) composed of three distinct sub-structures, the fibrillar center (FC) and dense fibrillar component (DFC), wherein rRNA genes are transcribed and start their processing, and the granular component (GC) where processing proceeds with the assembly of ribosomal proteins to rRNA to form preribosomal particles [16,120]. NPM1 is highly abundant within the GC where it exerts its functions related to ribosome biogenesis and cellular stress responses [15]. Many studies proposed NPM1 as a “nucleolar hub” because of its roles in nucleolar assembly and also on the basis of the vast array of interactions that occur mostly with nucleolar proteins [15]. Although depletion of NPM1 has been reported to result in disruption of nucleolar structure [121], its role in the architectural organization of nucleoli has not been fully elucidated until recently. A great effort to shed light on this matter has been made, primarily by the Kriwacki group [17,33,122]. Indeed, they elegantly showed that NPM1 directly participates in the organization of the liquid–liquid phase separation (LLPS) that stands at the basis of nucleolar architecture via a multimodal mechanism, involving interactions with nucleolar proteins and rRNA, but also by interacting with itself [17,33]. Indeed, NPM1 was shown to be able to form dense, liquid-like droplets through the engagement of R-rich motifs derived from protein partners. Such motifs have features in common or coincide with canonical NoLS sequences, suggesting a general mechanism for nucleolar localization mediated by NPM1 [33]. In addition, it was shown that NPM1 is also able to phase-separate with rRNA, in the presence of its folded C-terminal nucleic acid-binding domain, in cooperation with the adjacent disordered basic tract [33]. Importantly, both these structural features were required for proper phase separation of NPM1 with these two classes of nucleolar macromolecules and also for NPM1 localization within mammalian nucleoli [17,33]. These heterotypic-LLPS mechanisms are mainly driven by electrostatic interactions occurring between negatively charged A-tracts and core domain on NPM1 and positively charged R-motifs on proteins, and between positively charged basic tracts and the CTD on NPM1 and rRNAs, suggesting that they are mutually compatible within NPM1 pentamers. Importantly, it was observed that heterotypic processes are complemented by homotypic ones, i.e., processes involving interactions between different domains of NPM1, likely working concurrently to determine the liquid-like features of GC [17]. Homotypic-LLPS processes were found to be promoted by molecular crowding and due to electrostatic interpentamer interactions, resulting in increasingly interconnected NPM1-NPM1 networks and reduced NPM1 mobility [17,122]. It was thus proposed that NPM1′s different mixtures of homotypic and heterotypic interactions might facilitate the association of ribosomal proteins with rRNA and exit of ribosomal subunits from nucleoli. Accordingly, heterotypic associations might be central nearby the FC and DFC, where rRNA and ribosomal proteins are assembled into preribosomal particles, while homotypic interactions would dominate when preribosomal subunits exit the nucleolus [17]. Recently, a role for the nucleolar protein SURF6 (Surfeit locus protein 6) within this context was also investigated. SURF6 directly interacts and colocalizes with NPM1 in the GC and displays multiple R-motifs [33,123]. SURF6 was hypothesized to modulate the heterotypic vs homotypic interactions played by NPM1 in liquid-like droplets towards the former [122]. These findings suggested that interpentamer NPM1 interactions may respond to changes in the surrounding nucleolus content, coordinating with other nucleolar actors. To integrate these findings, a very recent work reported that NPM1 phase separates in vitro also with p14ARF (see previous paragraph) forming a condensed phase in which both proteins are reduced in their mobility [124]. In these networks NPM1 forms, through its N-terminal domain, a rigid scaffold of immobilized pentamers, while the central IDR and the C-terminal domain exhibit relative mobility, suggesting that they may be involved in sensing changes in the surrounding environment [124]. Further investigations will be needed to better understand the architectural structure of nucleoli within cells and these intricate processes that are essential to cellular homeostasis.

## 4. Inhibition

NPM1 is a multifunctional protein involved in several cellular mechanisms that depend on its complex interaction network and subcellular localization [1,2,3]. NPM1 overexpression, chromosomal translocation or mutations of the *NPM1* gene, are associated with many cancer types, both solid and hematological malignancies. The contribution played by NPM1 overexpression in solid tumors is likely associated with enforced ribosome synthesis and export, stimulation of DNA repair following oncogene activation and reduced apoptotic response due to p14ARF and p53 inhibition. In hematological tumors with translocations of the *NPM1* gene, the contribution of NPM1 stands in its oligomerization properties that facilitate the constitute activation of the kinase partners in the different chimeras. Finally, in AML with NPM1c+ expression, the capacity of NPM1c+ to delocalize in the cytosol crucial protein partners appears to be key to cellular transformation. In all cases, NPM1 is considered a promising therapeutic target [118,125] and many compounds, both natural and synthetic molecules, have been identified and are being developed as investigational drugs [118,125]. These molecules may target distinct structural domains of NPM1, thus affecting the ability of the protein to self-interact or interact with either protein or nucleic acid partners, with various consequences on its functional roles. Hereafter we provide an update of the most investigated or promising compounds and what is currently known on their effectiveness in targeting NPM1 in cancer.

### 4.1. Natural Compounds

(+)-Avrainvillamide (hereafter AVA) is an alkaloid isolated from a strain of *Aspergillus sp.* that has displayed antiproliferative effects in a panel of different cancerous cell lines [126,127]. AVA can specifically alkylate Cys275 of NPM1 CTD, thus forming with the latter tight complexes [127]. In vitro experiments highlighted that AVA also binds the mutant form of NPM1, with a higher affinity with respect to wild-type, probably due to the unfolded status of NPM1c+ CTD, and partially re-localizes it within the nucleoli of OCI-AML3 cells, a useful model cell line of AML carrying NPM1c+ [128]. Conversely, AVA did not induce nucleolar displacement of the wild-type protein, suggesting that AVA could function as a surrogate for the compromised NoLS in NPM1c+ [128]. These results were later confirmed in AML primary cells bearing *NPM1*-mutations [129]. Treatment of OCI-AML3 cells with AVA and a synthetic analog induced proteosomal degradation of NPM1c+ and monocytic differentiation of cells together with increased phagocytic activity [129].

Another natural compound that has shown efficacy against NPM1c+ is Oridonin [130]. This is a plant-derived tetracycline diterpenoid that holds anticancer and antiproliferative activities in a number of tumors, inducing apoptosis in cancer cell lines of diverse origin [131]. Oridonin treatments of OCI-AML3 cells dose-dependently inhibited viability and caused NPM1c+ translocation to the nucleus, likely due to CRM1 (Chromosomal Region Maintenance 1) nuclear accumulation, together with unchanged total NPM1 protein levels. It was suggested that NPM1c+ translocation might facilitate oridonin-induced apoptosis, due to increased p53 levels and caspase-3 activation [130].

Deguelin is a rotenoid isolated from several plant species of the *Leguminosae* family, whose antitumorigenesis and antiproliferative activities have been observed both in vitro and in vivo in various cancer types (i.e., lung, prostate, gastric, and breast), mostly by inhibiting cell proliferation and inducing apoptosis, but also by exerting antiangiogenic effects [132,133]. Deguelin has been reported to function as a selective silencer of the mutant form of NPM1 by greatly downregulating NPM1c+ protein levels, without affecting those of wild-type NPM1. These findings were observed in patient-derived primary blasts bearing *NPM1* mutations and in mouse xenograft models, in addition to the usual OCI-AML3 model cell line [134,135]. In particular, high-dose deguelin treatment was cytotoxic and induced apoptosis with concomitant caspase-6 and 8 activation in a more pronounced way in NPM1c+-expressing cells, as compared to wt-NPM1 cells [134]. On the other hand, deguelin treatments at a lower and nontoxic dosage led to selective differentiation of NPM1c+ cells, as shown both in vitro and in vivo [134,135].

Even if little is known about the mechanistic basis, NPM1c+ levels were observed to be downregulated also by the phenolic flavonoid epigallocatechin-3-gallate (EGCG) which induced apoptosis in IMS-M2 cells harboring *NPM1* mutations [136], in line with the antitumor activity found in other studies [137].

### 4.2. Synthetic Compounds

NPM1 is a protein that effectively oligomerizes, in vitro and in vivo, forming crown-shaped pentameric rings, due to the tight self-association of its N-terminal domain. It has been widely reported that the monomer/oligomer equilibrium of NPM1 is functional to its multifaceted roles. Therefore, interfering with the ability of NPM1 N-terminal domain to self-associate has been considered an amenable option to inhibit NPM1 specific roles within cancer cells. Consequently, in the past years, several NPM1 interface disruptors have been identified and experimentally tested in a panel of tumors.

One of the most investigated compounds is the small synthetic molecule NSC348884 (N,N,N′, N-tetrakis[(5-methyl-1H-benzimidazol-2-yl)methyl]ethane-1,2-diamine) designed to bind a hydrophobic pocket at the interface of NPM1 monomers in the pentamer, thus shifting the NPM1 monomer/pentamer equilibrium towards the monomer [138]. Oligomeric state disruption by NSC348884 compromised NPM1 chaperone activities while also affecting its protein interactions [138]. Consequently, NSC348884 treatment was shown to inhibit cell growth and to promote apoptosis, in a dose-dependent manner, in several cancer cell lines, including prostate and colorectal carcinomas, lymphoma [138] and hepatocellular carcinoma [139]. Elevated levels of p53 and its phosphorylation in conjunction with increased p21 levels were observed in all cases [138,139]. Overall, these findings suggest that this small molecular inhibitor might counteract the antiapoptotic activity of overexpressed NPM1, which may consist of decreased p53 levels as well as activation by its direct binding of or by indirectly promoting p53 degradation via the p14ARF-MDM2 axis [140,141]. Interestingly, Balusu and colleagues [142] have shown that AML NPM1c+-harboring cells are more sensitive to NSC348884 toxicity given greater effectiveness in disrupting NPM1 oligomerization. A higher apoptosis rate was found in NPM1c+-cells in comparison with wt-NPM1 cells [142]. However, recent findings suggested that NSC348884 might not exert its cytotoxic activity through inhibition of NPM1 oligomerization given that disruption of NPM1 oligomers, even in NPM1c+-expressing cells, was not observed [143]. This conflicting evidence needs to be further elucidated.

NPM1 oligomerization can also be impaired by RNA aptamers [144], single-stranded RNA or DNA molecules that bind and inhibit with high affinity and specificity target molecules. They are mostly isolated through “systematic evolution of ligands by exponential enrichment” (SELEX) and, to date, some of them have been approved for clinical use [145]. In order to inhibit NPM1 antiapoptotic activities, the 1A1 RNA aptamer and its truncated 40-mer form were selected and found to target the central acidic region (residues 114–187) of NPM1. Surprisingly, they affected NPM1 oligomerization both in vitro and within several cancer cell lines [144]. NPM1 monomer accumulation, its displacement in the nucleoplasm, and an increased apoptotic rate, due to p14ARF nuclear accumulation together with raised p53 and p21 expression levels [144], were observed, similar to what already described with NSC348884 [138].

Among synthetic compounds, the potent radiosensitizing molecule YTR107 (5-((N-benzyl-1H-indol-3-yl)methylene)pyrimidine-2, 4, 6 (1H, 3H, 5H)-trione)) was shown to bind NPM1 at its N-terminal core domain (residues 1–122) and to promote its monomerization [45,146]. It was suggested that YTR107 radiosensitization is mediated by NPM1 involvement in the homologous recombination (HR) repair machinery to resolve DNA double-strand breaks (DSBs) [147]. Upon phosphorylation at Thr199, NPM1 was shown to be recruited to irradiation-induced foci (IRIF) by binding ubiquitinated chromatin in an RNF8/RNF168-dependent manner [147,148]. YTR107 analogs have been also developed showing increased effectiveness in a panel of cancer cell lines, including NPM1c+ OCI-AML 3 cells [148].

CIGB-300, a synthetic cyclic peptide fused at its N-terminus to a cell-permeable peptide derived from the HIV-Tat protein, was shown to bind NPM1. This compound was designed to interfere with protein kinase CK2 phosphorylation by interacting with CK2 target sequences [149]. CIGB-300 exhibited wide anticancer properties in vitro, in tumor animal models, and also in cancer patients [149,150,151]. Notably, CIGB-300 has been subjected to several phase I clinical trials and has entered phase II [151,152]. Interestingly, CIGB-300 was shown to bind NPM1 and to inhibit CK2-mediated phosphorylation of NPM1 at Ser125 inducing damage to the nucleolar architecture and massive apoptosis [149], in line with the putative implication of NPM1 phospho-Ser125 in the maintenance of the nucleolar assembly, in ribosome biogenesis, and in cytokinesis [153,154,155].

NPM1 binds nucleic acids, including rDNA G-quadruplex sequences both in vitro and in vivo, through its C-terminal domain and depending critically on its folded state [36,42]. TmPyP4 (tetra-N-methyl-pyridyl porphyrin), a cationic porphyrin able to bind G-quadruplex DNA structures with high affinity, has been investigated by several groups for its anticancer and antiproliferative activities [156,157,158,159]. As to NPM1, TmPyP4 was shown to effectively displace NPM1 from nucleoli in both wild-type and mutant NPM1-expressing AML cells, without affecting NPM1 total content [42,160]. Interestingly, TmPyP4 was found to be more toxic for wt-NPM1 OCI-AML2 than for NPM1c+ OCI-AML3 cells, possibly due to the p14ARF NPM1c+-induced cytoplasmic delocalization and degradation of p14ARF, which delayed p53 activation [68,160].

Given the numerous proteins bound by NPM1, inhibition of these interactions might affect its role as a “hub” protein within the nucleoli and result toxic [15,161]. As already outlined in the previous section, a large surface in the N-terminal domain of NPM1 serves as a docking site for proteins carrying a NoLS rich in positively charged residues [26,113,115]. In the past years, NPM1 has been identified as one of the targets of the synthetic pseudopeptide NucAnt N6L (hereby N6L), a pro-apoptotic compound able to exert antiproliferative activity and to inhibit tumor growth also in vivo, that has already completed Phase I/IIa clinical trials for different solid tumors [162,163,164]. Structurally, N6L consists of a multimeric pseudopeptide highly enriched in positively charged residues, hence resembling the properties of a NoLS [162,165]. Recently, N6L was shown to bind NPM1 to a high-affinity site in the NPM1 N-terminal domain and to a lower-affinity site in the central domain. Furthermore, N6L was able to displace NPM1 interactors, thus interfering with NPM1 protein–protein associations [165]. N6L activity was tested on wt-NPM1 and NPM1c+-expressing AML cells, OCI-AML2 and OCI-AML3, respectively, and shown to colocalize with nucleolar NPM1 and cytoplasmic NPM1c+, respectively [165]. N6L treatment consistently reduced OCI-AML2 cell growth through p53-mediated apoptosis, while its effect was less appreciable in OCI-AML3 cells, due to delayed p53 activation. However, N6L sensitized both AML cell lines when administered together with standard chemotherapeutics, either doxorubicin or cytarabine or both [165].

In recent years, a number of compounds targeting the interaction between NPM1 and APE1 (Apurinic/apyrimidinic endonuclease 1) have been identified [166]. APE1 is a major enzymatic actor in the Base Excision Repair (BER) pathway and NPM1 is able to modulate several functions of APE1, from its subcellular localization to its endonuclease activity and its interaction network [69]. APE1 is, consequently, wrongly delocalized within the cytoplasm in *NPM1*-mutated AML cells, leading to BER impairment and increased sensitivity to genotoxins [69]. Three bioactive compounds (spiclomazine, fiduxosin and SB206553), selected through high-throughput screening, inhibited NPM1-APE1 association and displayed antiproliferative activity in several cell lines [166,167].

NPM1 contains two nuclear export signals (NES) within its N-terminal domain that are recognized by the nuclear exportin CRM1 (also denoted as XPO1). In NPM1c+, a novel NES in the protein C-terminus is formed which reinforces those already present and determines cytoplasmic export [41]. Blocking the CRM1 export mechanism may be a useful strategy for preventing NPM1c+ cytoplasmic translocation. The antifungal compound Leptomycin B (LMB) was shown to target CRM1 in its NES-binding groove, but failed Phase I clinical trials due to severe dose-limiting toxicity [168]. Therefore, novel small selective inhibitors of nuclear export (SINE) were developed, including the orally bioavailable Selinexor (also named KPT-330; ((Z)-3-(3-(3,5-bis(trifluoromethyl)phenyl)-1H-1,2,4-triazol-1yl)-N’-(pyrazin-2-yl) acrylohydrazide) that has been explored in clinical studies in both solid and hematological malignancies [168,169]. Leukemic cell lines treated with Selinexor were shown to undergo p53-dependent apoptosis and differentiation [170], while Selinexor administration prolonged survival in a leukemia mouse model [171]. Recently, Gu and collaborators [71] demonstrated that Selinexor treatment of a patient-derived xenograft mouse model of *NPM1*-mutated AML restored NPM1c+ nuclear localization and induced monocytic/granulocytic terminal differentiation, which was likely disrupted by NPM1c+-induced delocalization of the myeloid transcription factor PU.1 [71]. Brunetti and colleagues [172] obtained similar results by associating NPM1c+ nuclear re-localization with the downregulation of *HOX* genes and consequent differentiation of AML cells [172]. Of course, antileukemic effects exhibited by Selinexor may not be attributed solely to the re-localization of mutant NPM1, even if this event was shown to be sufficient to create a therapeutic vulnerability [71,172]. In addition, a synergistic effect in AML therapy was observed by several groups when Selinexor was combined with other chemotherapeutic agents (i.e., cytarabine, fludarabine, daunorubicin) [173,174,175]. Finally, a new generation of SINE inhibitors has been developed, exhibiting promising results in preclinical tests in leukemia [176,177]. On the base of these interesting findings, further investigations are needed with the aim to identify more specific SINE compounds.

## 5. Conclusions

NPM1 is a protein endowed with many crucial functions that are exerted in different cell compartments through the interaction with protein and nucleic acids partners. Though the protein has been studied since the eighties of the last century, the interest in NPM1 has literally exploded after the discovery of AML mutations in 2005. Since then, we have learned a lot about the physio-pathological features of NPM1. From a structural standpoint, a number of contributions shed light on the interaction of NPM1 domains with proteins and nucleic acids. Importantly, more recent studies are also starting to clarify the behavior of the protein as a whole, which implicates to take into account and describe, at once, the contribution played by both NPM1 homotypic and heterotypic interactions. Such exciting studies are starting to shed light on the role played by NPM1 in nucleolar architecture by its capacity to promote LLPS. A number of compounds have been identified that influence NPM1 oligomeric status, interfere with its association to nucleoli, inhibit its protein–protein interactions, prevent its cytosolic translocation and some of them are undergoing clinical trials. As to now, these trials are focused on the treatment of hematologic malignancies and other types of cancer, however, the contribution that NPM1 plays in the life cycle of many viruses suggests the investigational use of NPM1 inhibitors also as antivirals. Furthermore, given the pervasive nature of this protein in a variety of cellular processes, it is not unreasonable to hypothesize that future research will disclose a yet unknown role for NPM1 in other pathologies. The very recent discovery of novel mutations in the NPM1 central domain that cause dyskeratosis congenita is a very good example of this line of thinking and demonstrates that NPM1 research, after more than 30 years from its discovery, is still very lively.
